# Exploring the psychological impact of long COVID: symptoms, mechanisms, and treatments

**DOI:** 10.3389/fpsyt.2025.1555370

**Published:** 2025-08-19

**Authors:** Shi Shen, Xin Zhao, Jianxin Pei, Bijue Wang, Jingjing Hou, Ru Chai, Yiqiong Guo, Feiyu Li, Jian Hao, Zhonglan Wu

**Affiliations:** ^1^ Infection Control Office, Xi’an Public Health Center (Xi’an Emergency Medical Center), Xi’an, Shaanxi, China; ^2^ Virology Department, Ningxia Hui Autonomous Region Center for Disease Control and Prevention, Yinchuan, Ningxia, China; ^3^ Endemic Disease Control Division, Lianhu District Center for Disease Control and Prevention, Xi’an, Shaanxi, China; ^4^ Occupational Health Department, Weiyang District Center for Disease Control and Prevention, Xi’an, Shaanxi, China; ^5^ Preventive Healthcare Department, Yanta District Traditional Chinese Medicine Hospital, Xi’an, Shaanxi, China

**Keywords:** long COVID, psychological symptoms, mechanisms, epidemiology, interventions

## Abstract

Long COVID (LC) refers to a multisystem condition that persists after infection with severe acute respiratory syndrome coronavirus 2 (SARS-CoV-2), the virus responsible for coronavirus disease 2019 (COVID-19). In addition to physical symptoms, the psychological impact is particularly pronounced. This review summarizes the manifestations, potential mechanisms, epidemiological characteristics, and current interventions related to psychological disorders in LC. Drawing on domestic and international literature, it highlights anxiety, depression, cognitive dysfunction, and post-traumatic stress disorder (PTSD) as the primary psychological symptoms. These symptoms may be associated with neuroinflammation, immune abnormalities, vascular dysfunction, and psychosocial stress. Although research in this area is still developing, psychotherapy, pharmacotherapy, neuromodulation, and lifestyle interventions show promise as treatment approaches. This review aims to provide insights that can inform future research on clinical treatments and psychological care for individuals with LC.

## 1 Introduction

Since the outbreak of coronavirus disease 2019 (COVID-19) in 2019, hundreds of millions of people worldwide have been infected. Although the initial symptoms often subside within weeks for the majority of patients, 10%–20% of people encounter enduring or recurring symptoms, ultimately progressing to long COVID (LC) (1). A meta-analysis reported that 45% of COVID-19 survivors continue to experience unresolved symptoms after recovery (2). LC is a chronic, infection-related condition that manifests post-COVID-19, last for a minimum of three months, and is distinguished by a cycle of ongoing relapse and remission or escalating deterioration. It may impact one or more organ systems (3). Although COVID-19 is primarily considered a respiratory disease, it also invades the central and peripheral nervous systems, making it closely associated with various neuropsychiatric manifestations (4, 5). Additionally, it is often associated with significant psychological symptoms: around 30% of patients with LC have psychological symptoms such as exhaustion, while nearly 20% endure psychological disorders like despair, anxiety, and post-traumatic stress disorder (PTSD) (6, 7). Individuals with LC often experience high levels of anxiety and depression, which may result not only from persistent physical symptoms but also as a direct consequence of the initial SARS-CoV-2 infection (8). A systematic review and meta-analysis found that the prevalence of at least one symptom during follow-up was 52.6% among hospitalized patients and 34.5% among non-hospitalized patients (2). According to a study conducted in Wuhan, China, more than 55% of previously disorders COVID-19 patients continued to report symptoms two years after discharge (9). Another systematic review and meta-analysis indicated that three years after infection, the most common unresolved psychological symptoms in COVID-19 patients were fatigue and anxiety. This population tends to have poorer health-related quality of life (HRQoL), reduced physical function, more post-discharge psychological symptoms, and increased healthcare utilization, thereby imposing a substantial burden on both individuals and national healthcare systems (10).

Since the onset of the COVID-19 pandemic, the public has been facing unprecedented challenges and pressure related to psychological health (11). Psychological problems not only compromise patients’ quality of life and exacerbate physical illness but can also have significant economic and psychosocial implications (12). On December 7, 2022, China optimized its COVID-19 prevention and control policies, and from January 8, 2023, reclassified COVID-19 as a Category B infectious disease managed with Category B measures (13, 14). Since then, addressing LC and supporting recovery have become pressing issues in China. In a *Lancet* article entitled “China needs a scientific long COVID recovery-support platform,” Chinese scholars pointed out that there is currently no publicly accessible and authoritative platform in China to explain LC symptoms. As a result, the public lacks access to reliable scientific information, leading to insufficient understanding and awareness of LC. The absence of an official, credible source of information or support platform for LC patients may prolong or worsen their symptoms and contribute to psychological distress due to a lack of timely guidance (15).

To date, much of the focus on COVID-19 has centered on physical symptoms and outcomes. However, there is a growing recognition of the pandemic’s impact on psychological health and an urgent need for appropriate psychological health services to provide psychological support. Therefore, systematically reviewing the research progress on psychological symptoms in LC patients and exploring underlying mechanisms and intervention strategies is of great importance for improving psychological health outcomes and quality of life.

## 2 Stigmatization and psychological disorders in LC

### 2.1 Stigmatization

The stigmatization of psychological health issues can be subdivided into two dimensions: social stigma and illness-related shame. Social stigma refers to the negative attitudes and discrimination commonly faced by individuals suffering from health conditions and unexplained physical symptoms (16). During the COVID-19 pandemic and in the context of LC symptoms, the impact of social stigma on psychological health has been widely documented (17–19). Illness-related shame triggered by COVID-19 and LC rarely exists in isolation and is often accompanied by other forms of stigma. These stigmatizing experiences adversely affect individuals’ physical health and quality of life. The impact of social shame surrounding psychological health issues is a contributing factor to the deterioration of psychological well-being among LC patients. Shame can inhibit individuals from seeking help and sharing their experiences, potentially exacerbating feelings of isolation and distress (20). The spectrum of psychological problems associated with LC complicates recovery and intensifies the stigma related to psychological health (21). To reduce stigma and shame faced by individuals with long COVID, health education targeting the sources of social stigma and efforts to increase public awareness and understanding of the condition may represent an effective strategy (22).

### 2.2 Anxiety and depression

Anxiety and depression are the most common psychological disorders among LC patients (23). Anxiety symptoms typically include persistent worry, tension, irritability, inability to stop or control worrying, and feelings of nervousness and anxiety, which are among the most frequently reported symptoms. Depression primarily manifests as fatigue or lack of energy, low mood, sadness, or hopelessness, and in some cases, suicidal ideation (24). Some patients may also experience feelings of despair and diminished self-worth. Survivors exhibiting higher levels of anxiety and depression tend to more frequently express fear of COVID-19-related sequelae and report more symptoms post-discharge, especially fatigue and sleep disturbances (25, 26). A large body of literature has revealed that the challenges faced by COVID-19 survivors extend beyond physiological aspects, encompassing psychological stressors such as uncertainty related to the illness, social isolation, and financial burdens during recovery. Anxiety and depression symptoms may persist even after COVID-19 recovery, lasting up to 24 months (27).

### 2.3 Post-traumatic stress disorder

PTSD refers to a group of conditions triggered by trauma or other life stressors. COVID-19-related PTSD can result from both direct effects (the illness itself) and indirect consequences (living under stress, uncertainty, and changes in daily life), with PTSD symptoms potentially exacerbating other psychological disorders (28, 29). COVID-19 patients, particularly within months after hospital discharge, may experience PTSD symptoms (30). Some patients develop PTSD following intensive care treatment for severe COVID-19 or after losing loved ones. PTSD symptoms in LC patients can manifest in various ways, with commonly reported symptoms including intrusive thoughts related to the traumatic experience of COVID-19, increased anxiety, emotional numbness, and hyperarousal (31, 32). These symptoms significantly impair patients’ daily functioning and psychological health (33).

### 2.4 Fatigue

Fatigue is one of the most common symptoms of LC and can be defined as a decline in physical and/or mental performance caused by alterations in central, psychological, and/or peripheral factors related to COVID-19 (34). It is mainly characterized by fatigue that is not significantly relieved by rest, often accompanied by heterogeneous symptoms such as generalized body, muscle, and joint aches, mood disturbances, and sleep difficulties. Physical and laboratory examinations usually show no obvious abnormalities (35). While fatigue is a common and unavoidable symptom in daily life, LC patients experience a markedly increased burden of fatigue (36). Persistent fatigue is often associated with other symptoms such as dyspnea and cognitive impairment, which significantly impact the affected individuals’ quality of life and functional status (37).

### 2.5 Sleep disorders

Sleep disorders have become a major problem among LC patients. The types of sleep disturbances reported by LC patients are diverse, including insomnia, nightmares, excessive daytime sleepiness, and disrupted sleep architecture. Insomnia is one of the most common symptoms in LC patients, typically presenting as difficulty initiating sleep, frequent nocturnal awakenings, or early morning awakenings. Excessive daytime sleepiness is also a frequent neuropsychiatric manifestation; some LC patients experience difficulty maintaining wakefulness during the day, and some may meet diagnostic criteria for idiopathic hypersomnia or type 2 narcolepsy according to international sleep disorder classifications (38). LC patients often complain of poor sleep quality and feeling exhausted upon waking, with insufficient restoration of energy (39). Moreover, the relationship between sleep disorders and other neuropsychiatric symptoms such as anxiety and depression is well established. Sleep problems may be not only a direct consequence of viral infection but also closely related to psychological health (40).

### 2.6 Cognitive dysfunction

**Figure 1:**
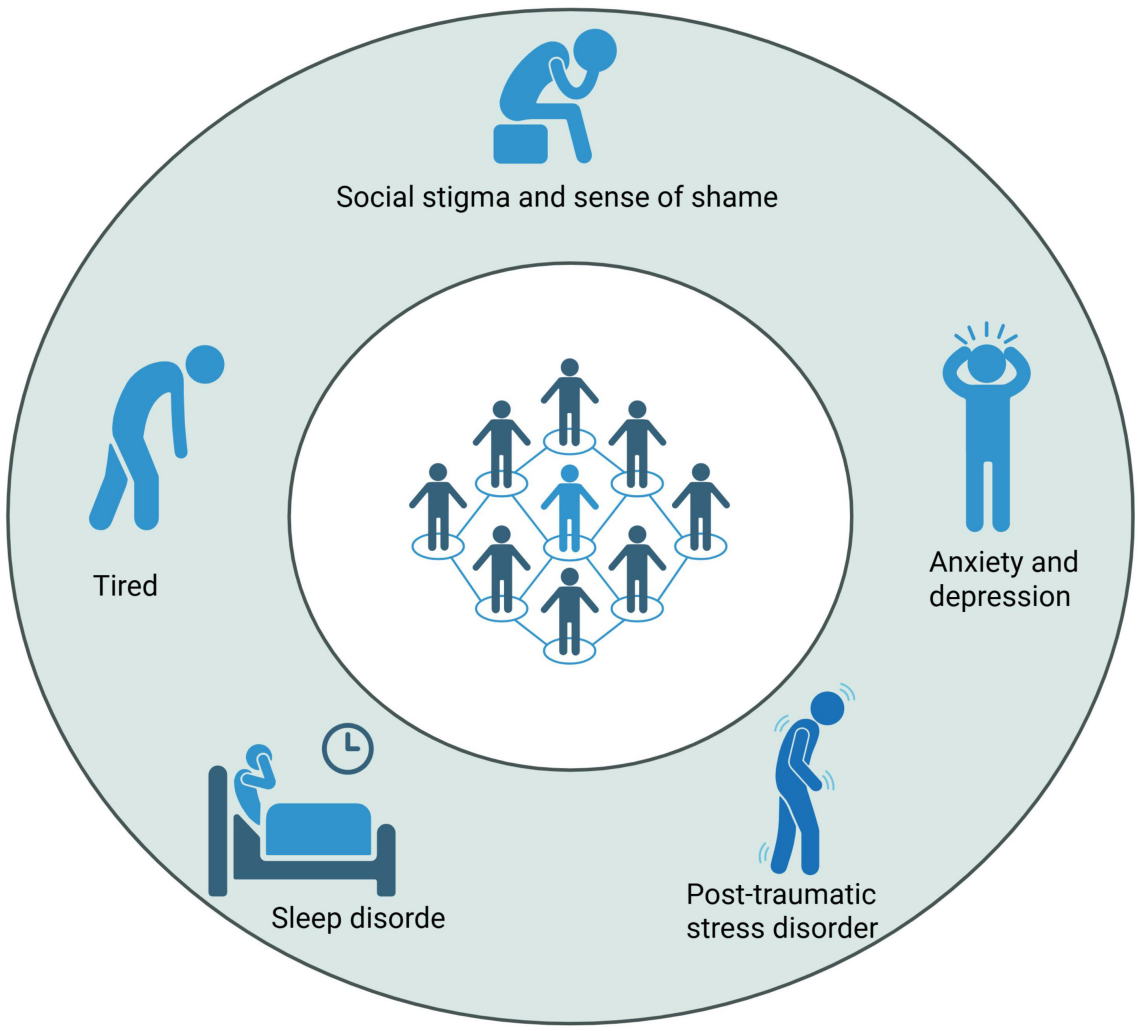
Psychological symptoms of LC.

Cognitive dysfunction in LC patients is characterized by a range of symptoms, including memory decline, attention deficits, executive dysfunction, and reduced language fluency, often accompanied by psychological distress (41). A case-control study using the Addenbrooke’s Cognitive Examination III screening test found that the overall cognitive function of LC patients was significantly lower than that of recovered controls. Additionally, 48% of LC patients exhibited episodic memory deficits, with 27% showing overall cognitive impairment, particularly in attention, working memory, processing speed, and language fluency (41). Cognitive symptoms are often accompanied by other behavioral manifestations such as anxiety, depression, and sleep disorders, which collectively exacerbate the cognitive burden (42). Cognitive dysfunction related to LC is relatively common and persistent, affecting individuals across all age groups (43). The duration of cognitive impairment varies among patients; some experience symptom improvement after several weeks, whereas others suffer long-term cognitive problems that severely impact daily life and work performance (44, 45) (**Figure 1**).

## 3 Mechanisms of psychological disorders in LC

### 3.1 Direct viral invasion

Studies suggest that SARS-CoV-2 may persistently reside in tissue reservoirs within LC patients, triggering immune responses that contribute to symptom persistence (46–48). SARS-CoV-2 can enter the central nervous system (CNS) via multiple routes, including trans-synaptic transfer, the olfactory nerve, the blood-brain barrier, and hematogenous spread, leading to direct neuronal injury (49). This direct damage may contribute to the development of neuropsychiatric symptoms such as anxiety and depression, which are commonly observed in LC patients. Moreover, the continued presence of viral particles or remnants within the CNS may exacerbate inflammation, causing demyelination and neurodegenerative changes (50, 51), which in turn may trigger neuroinflammation and mental health issues (52, 53).

### 3.2 Immune dysregulation

Research has demonstrated that COVID-19 induces an overactive immune response, with increased cytokine release associated with various psychiatric disorders (54, 55). Depression and COVID-19 share similar immune functional patterns, particularly a pro-inflammatory state characterized by elevated cytokines including IL-6, TNFα, and IL-1β (56). Immune activation can impair neuronal function and affect brain plasticity (56). Prolonged immune activation is likely to disrupt brain function, especially in regions controlling emotion and cognition, such as the prefrontal cortex and hippocampus, leading to symptoms including depression, anxiety, and cognitive impairment. Recent studies support the long-term impairment of cognitive functions—especially executive function—and their association with inflammatory markers (57).

### 3.3 Neuroinflammation

Neuroinflammation results from the interaction between the virus and the immune system, causing the release of pro-inflammatory cytokines that affect brain function and emotional regulation. The immune response to SARS-CoV-2 may directly impact psychological health (58, 59). This inflammatory response can persist long after the acute phase of infection, contributing to chronic psychological issues observed in LC patients (60). Neuroinflammation is a prominent feature of LC, with studies demonstrating significant *in vivo* neuroinflammatory responses in LC patients (61). Widespread neuroinflammation in LC patients, which was associated with cognitive decline and structural brain changes such as cortical thinning and reductions in gray matter volume over time (58, 59). This neuroinflammatory state is thought to be driven by the immune response to the initial viral infection, leading to microglial activation and release of pro-inflammatory cytokines that can damage neuronal cells and disrupt synaptic function (62). Elevated levels of inflammatory markers in the cerebrospinal fluid of LC patients further support the role of neuroinflammation in the pathogenesis of neurological symptoms (63).

### 3.4 Vascular dysfunction

Vascular dysfunction contributes to the development of mood disorders because the brain’s ability to regulate emotions is closely linked to its vascular health. SARS-CoV-2 may directly affect the vascular system, causing endothelial dysfunction and microvascular injury, thereby exacerbating psychological disorders (64). Studies indicate that SARS-CoV-2 can trigger systemic vascular problems, especially in severely infected patients, where endothelial dysfunction and microvascular pathology are common. Inflammation-induced signaling caused by SARS-CoV-2 and endothelial cell dysfunction promotes a hypercoagulable state (65, 66). Microvascular thrombosis impairs oxygen exchange, and widespread cellular hypoxia may contribute to LC symptoms such as sleep disturbances, anxiety, and depression (67, 68).

### 3.5 Hypothalamic-pituitary-adrenal axis dysregulation

The physiological mechanisms of LC, particularly psychological disorders, may be closely related to dysregulation of the HPA axis. This dysregulation is a key neuroendocrine aspect of the stress and inflammatory response. Research shows that the HPA axis plays a critical role in the human stress response. SARS-CoV-2 can induce increased systemic inflammation, especially elevated levels of pro-inflammatory cytokines such as IL-6 and TNF-α, which may lead to hyperactivation of the HPA axis. Activation of the HPA axis results in the release of glucocorticoids, which are crucial for regulating immune responses and maintaining homeostasis (69). Cortisol levels influence brain regions involved in emotional regulation, and sustained high cortisol may be associated with the development of psychological symptoms such as anxiety and depression, which may partially explain the presence of such symptoms in LC patients (70, 71).

### 3.6 Psychosocial stress

**Figure 2:**
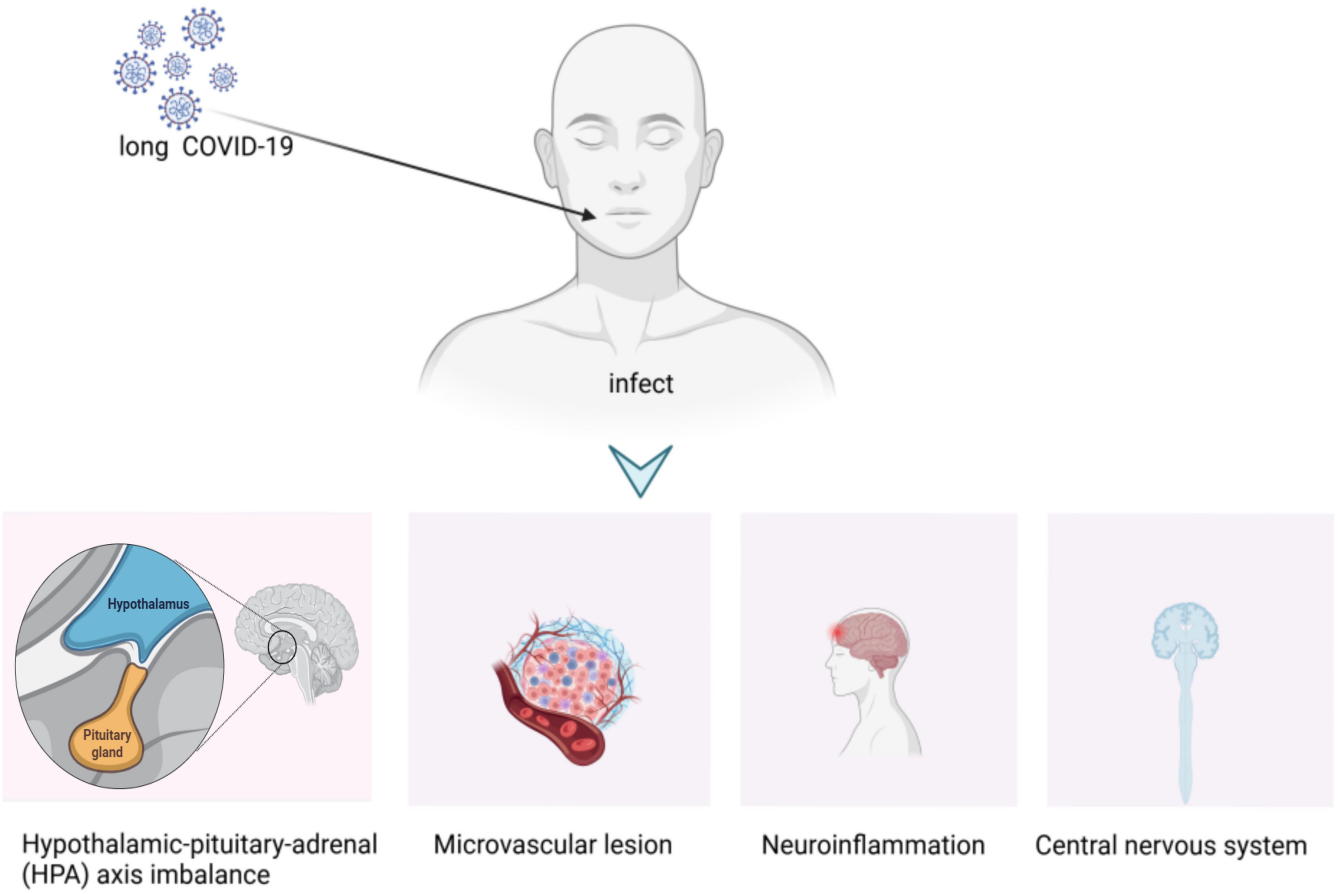
Physiological mechanisms of organ damage in LC.

Beyond biological mechanisms, psychosocial factors also play a significant role in the psychological symptoms experienced by LC patients (72). The experience of COVID-19 itself can cause substantial psychological stress, which may trigger or exacerbate pre-existing psychological issues. Studies show that individuals with a history of mental illness are at higher risk of developing new psychological problems post-infection, with factors such as social isolation, stigma, and stress related to coping with health uncertainty playing key roles (73, 74). The psychological impact of the COVID-19 pandemic, including fear of illness and disruptions to daily life, may contribute to disorders such as PTSD, anxiety, and depression (75, 76) (**Figure 2**).

## 4 Epidemiological characteristics of psychological symptoms in LC

### 4.1 Prevalence

The occurrence of psychological symptoms is significantly higher among LC patients worldwide (77, 78). Studies reveal that approximately 30% of LC patients exhibit psychological symptoms. One survey found that the vast majority of LC patients have experienced some form of social exclusion, with 95.4% encountering social stigma at least occasionally and 75.9% experiencing it frequently (79). Due to heterogeneity in study populations and neuropsychological assessments, prevalence estimates vary considerably. Two meta-analyses estimated the overall prevalence of cognitive impairment to range between 14% and 22% (35, 80). The prevalence of anxiety symptoms ranges from 6.8% to 47.8%; depression from 4.4% to 35.9%; PTSD from 13.0% to 42.8%; and sleep disorders from 4.4% to 50.0% (81). Recent studies indicate that a substantial proportion of LC patients exhibit PTSD symptoms, with an estimated over 49% meeting diagnostic criteria for PTSD (82). A systematic review integrating data from 165 studies involving 9,923,461 patients showed that among COVID-19 survivors, the prevalence rates for depression and anxiety were both 23%, and sleep disorders 45% (83) (**Table 1**). An online survey including 3,726 participants from 56 countries reported a 78.6% prevalence of sleep disturbances over more than seven months of follow-up, including insomnia (60%), night sweats (41%), awakening with breathing difficulty (36%), restless legs syndrome (18%), and sleep apnea (10%) (43). Variability in reported prevalence rates of psychological symptoms across studies may be attributed to differences in study design, assessment methods, sociocultural contexts, viral variants, and demographic characteristics. Furthermore, different systematic reviews and meta-analyses include varying ranges of literature, contributing to discrepancies in prevalence estimates. The duration of psychological symptoms in LC varies individually; most patients experience symptom improvement within months post-infection, but some persist beyond six months, even exceeding 24 months, with relapsing episodes (10).

**Table 1:** Main features of referenced studies on psychological symptoms associated with long COVID.

Study author (Year)	Sample size (n)	Age (Mean or range, years)	Sample source	Hospitalization status	Study design	Psychological symptoms
Tianqi Yang (2022) (6)	167	≥18	Electronic databases (PubMed and Web of Science)	Hospitalized	Systematic review and meta-analysis	Fatigue 27.5% (95% CI 22.4–33.3%), Sleep disorders 20.1% (95% CI 14.7–26.9%), Anxiety 18.0% (95% CI 13.8–23.1%), PTSD 14.6% (95% CI 11.3–18.7%), Depression 12.7% (95% CI 9.3–17.2%)
Lauren L. O’Mahoney (2022) (2)	735,006	3–74	Electronic databases (MEDLINE, Cochrane Library, Scopus, CINAHL, medRxiv)	Hospitalized, Non-hospitalized	Systematic review and meta-analysis	Hospitalized: Fatigue 28.4% (95% CI 24.7–32.5%), Sleep disorders 23.5% (95% CI 18.1–29.8%)
						Non-hospitalized: Fatigue 25.2% (95% CI 17.7–34.6%)
Na Zeng (2022) (17)	1,285,407	Not reported	Electronic databases (PubMed, Embase, Cochrane Library)	Hospitalized, Non-hospitalized	Systematic review and meta-analysis	Fatigue 28.7% (95% CI 21.0–37.0%), Depression 18.3% (95% CI 13.3–23.8%), PTSD 17.9% (95% CI 11.6–25.3%), Cognitive impairment 19.7% (95% CI 8.8–33.4%), Memory impairment 17.5% (95% CI 8.1–29.6%)
Masoud Rahmati (2025) (11)	142,171	36–86	Electronic databases (PubMed, MEDLINE [Ovid], CENTRAL, Web of Science, Scopus, Embase)	Hospitalized, Non-hospitalized	Systematic review and meta-analysis	Fatigue 11% (95% CI 6–20%), Insomnia 11% (95% CI 2–37%), Anxiety 6% (95% CI 1–32%)
Niloofar Seighali (2024) (83)	9,923,461	Not reported	Electronic databases (PubMed, Scopus, Embase, Web of Science)	Not reported	Systematic review and meta-analysis	Anxiety 23% (95% CI 20–26%), Depression 23% (95% CI 20–26%), Sleep disorders 45% (95% CI 37–53%)
Halwa Zakia (2023) (81)	281,684	–	Electronic databases (Scopus, PubMed, EMBASE)	Hospitalized, Non-hospitalized	Systematic review	Anxiety 6.8–47.8%, Depression 4.4–35.9%, PTSD 13.0–42.8%, Poor sleep quality, Sleep disorders/insomnia 4.4–50.0%, Cognitive impairment 73.2%
Lixue Huang (2022) (10)	1,192	57.0 (IQR 48.0–65.0)	COVID-19 hospitalized survivors discharged from Jin Yin-tan Hospital (Wuhan, China)	Hospitalized	Cohort study	Sleep disorders 25%, Anxiety 8%, Depressive symptoms 6%, PTSD 2%
Hernan F. Guillen-Burgos (2023) (27)	1,565	51.47 (SD 19.60)	Discharged patients from emergency, inpatient, ICU departments, teaching hospital, Barranquilla, Colombia	Hospitalized	Cohort study	Anxiety 16.55%, Depression 21.79%, PTSD 35.27%, Insomnia 23.86%
César Fernández-de-las-Peñas (2021) (25)	1,142	61.0 (SD 17.0)	Four public hospitals, Madrid, Spain	Hospitalized	Cross-sectional study	Anxiety 16.2%, Depression 19.7%, Poor sleep quality 34.5%
Víctor M. Serrano del Pueblo (2024) (41)	105	23–72	Neurology Dept., University Hospital Complex, Albacete, Spain	Hospitalized, Non-hospitalized	Cross-sectional study	Anxiety 58%, Insomnia 57%, Episodic memory deficits 48%, Depression 46%, Cognitive impairment 27%
Sana A. Khan (2023) (77)	457	33.86 (SD 13.58)	Psychiatry department, tertiary care hospital, Karachi, Pakistan	Hospitalized, Non-hospitalized	Cross-sectional study	Anxiety 32.1%, Depression 22.6%, Sleep disorders 25.4%, Fatigue/hypersomnia 77%
Han Thi Vo (2024, Vietnam) (82)	4,463	18–85	18 hospitals and health centers, Vietnam	Not reported	Cross-sectional study	PTSD >49.6%
Hannah E. Davis (2021) (43)	3,762	≥18	Online survey platform Qualtrics	Not reported	Cross-sectional study	Cognitive dysfunction 81.5% (95% CI 83.9–86.2%), Sleep disorders 78.6% (95% CI 84.0–79.9%), Post-exertional malaise 89.1% (95% CI 88.0–90.0%)

### 4.2 Risk factors

Among demographic factors, female sex is consistently identified as an important risk factor. Research shows that females are more likely than males to develop psychological symptoms such as anxiety and depression. A prospective cohort study on females with LC found that the proportion of females experiencing most physical symptoms and all psychological symptoms was higher than males (84). This difference may be attributed to social expectations, the psychological burden of caregiving roles typically assumed by women, and hormonal influences (20, 85). However, some studies have shown that anxiety symptoms in male LC patients are higher than in females, which may be due to the small male sample size and limited representativeness in that particular study (20), individuals with a history of psychological health disorders such as anxiety or depression have a significantly higher risk of developing psychological problems in LC (86). During the pandemic, older adults faced more severe physical symptoms, whereas younger individuals appeared to be at higher risk for poor psychological functioning (87). This may be related to the emotional advantages associated with older age, which declined during the COVID-19 pandemic but still persisted (20). COVID-19 may trigger or worsen common chronic conditions in the elderly, such as cardiovascular disease, respiratory illness, neurodegenerative diseases, and functional decline; they may also have experienced bereavement during the pandemic, leading to physical and psychological frailty (88). Some studies suggest that younger populations may be more susceptible to the cognitive and psychosocial impacts of COVID-19 (72), and compared with older adults, younger individuals may have poorer capacity to regulate negative emotions related to the pandemic (89). Moreover, lower educational attainment and socioeconomic status have been associated with increased risk of psychological problems after the COVID-19 pandemic (90). Clinically, survivors with a history of mental illness or substance use are particularly prone to anxiety and depression symptoms post-infection (91). Patients with severe COVID-19 symptoms, especially those requiring hospitalization or intensive care, have a higher risk of developing psychological symptoms, notably PTSD (92). The coexistence of chronic diseases exacerbates psychological distress, as individuals must cope with both physical and psychological health challenges (90). Furthermore, COVID-19-related stigma has been recognized as a novel risk factor, inducing anxiety and distress among survivors (93). Regarding environmental factors, the context of the COVID-19 pandemic has been a significant contributor to psychological symptoms associated with LC (94). The widespread social isolation and lockdown measures have increased feelings of loneliness and anxiety among various populations (95).

## 5 Measures to improve psychological symptoms of LC

### 5.1 Psychotherapy

Cognitive Behavioral Therapy (CBT) is a common psychotherapeutic approach that alleviates psychological distress by identifying and modifying negative thought patterns and behaviors (96). CBT is widely applied and has shown good efficacy in the intervention of psychological symptoms in LC (97, 98). Studies indicate that CBT can effectively reduce anxiety, depression, insomnia, and fatigue symptoms in LC patients (99, 100). By altering patients’ negative cognitive patterns, CBT helps them adopt a more positive attitude toward illness and recovery. Additionally, other psychotherapies such as mindfulness therapy (101), meditation, and relaxation training have demonstrated beneficial effects. However, it is important to avoid repeated recounting of traumatic experiences to prevent secondary trauma (102). For patients with severe psychiatric disorders, early psychiatric specialist intervention is recommended. Moreover, enhancing the inclusivity of social support is an important strategy to improve the psychological health of LC patients (103).

### 5.2 Pharmacotherapy

For patients with more severe symptoms, pharmacological treatment can complement psychotherapy (104). Pharmacological approaches to treating psychological symptoms in LC typically involve antidepressants and anxiolytics. Selective serotonin reuptake inhibitors (SSRIs) and other classes of antidepressants have been shown to effectively treat common depression and anxiety symptoms in this population (105). Short-term use of anxiolytics, such as benzodiazepines, may be helpful for patients with prominent anxiety symptoms (106). The link between inflammation and psychological health is well established, with elevated pro-inflammatory cytokines (e.g., IL-6 and TNF-α) implicated in the pathogenesis of mood disorders; thus, adjunctive anti-inflammatory treatments may be an important strategy for managing psychiatric conditions (107). Nevertheless, long-term use of medications should be cautious and conducted under professional medical supervision.

### 5.3 Neuromodulation therapy

Non-invasive brain stimulation (NIBS) plays a significant role in improving psychological symptoms in LC. Neuromodulation techniques such as transcranial magnetic stimulation (TMS), deep brain stimulation (DBS), and vagus nerve stimulation (VNS) have demonstrated efficacy in treating anxiety and depression and are applicable in LC health management (108). TMS, which uses magnetic fields to stimulate specific brain regions and enhance neuroplasticity, has been used to treat treatment-resistant depression. TMS effectively improves depressive symptoms and cognitive impairment in LC patients (109). Transcutaneous auricular VNS exerts anti-inflammatory, analgesic, and antidepressant effects, showing beneficial impact on fatigue syndrome in LC patients (110). These techniques modulate neural activity in targeted brain areas, thereby influencing neural circuits involved in emotion and physiological responses, and are particularly suitable for patients resistant to pharmacotherapy (111).

### 5.4 Hyperbaric oxygen therapy

HBOT involves treatment with 100% oxygen at pressures exceeding one absolute atmosphere (ATA), enhancing oxygen delivery to tissues, reducing inflammation, and promoting healing (112, 113). HBOT is used for various medical conditions, including trauma, hypoxia, infections, and certain chronic diseases. Recently, HBOT has been explored as a potential treatment for some LC-related symptoms (114). HBOT can alleviate several symptoms associated with LC, including depression, fatigue, and cognitive dysfunction (113, 115). Its safety and efficacy have been well established, making it a feasible option for LC treatment (116).

### 5.5 Lifestyle interventions

Lifestyle interventions have been identified as potential means to improve psychological symptoms associated with LC (117). Exercise and diet, as adjunctive treatments, play roles in alleviating psychological symptoms in LC patients. Moderate physical activity, especially aerobic exercise, helps improve mood and cognitive function (118). Maintaining regular sleep routines also benefits anxiety, depression, and stress (119). A balanced diet rich in antioxidants and anti-inflammatory compounds may help reduce neuroinflammation and alleviate psychological symptoms. Sleep disturbances related to LC are associated with inflammation, and nutrients such as zinc, vitamins C and D, and polyphenols may improve inflammation and sleep quality, thereby reducing symptoms (120). Coenzyme Q10 and alpha-lipoic acid, alone or combined with acetyl-L-carnitine as part of dietary interventions, have shown efficacy in reducing fatigue (121, 122). In summary, lifestyle interventions—including physical activity, sleep management, and nutritional support—are critical for mitigating psychological symptoms associated with LC.

### 5.6 Social support and rehabilitation programs

The establishment of social support systems is crucial for alleviating psychological symptoms in LC patients. Communities and families should provide emotional support to help patients cope positively with life’s challenges (123). Additionally, personalized rehabilitation programs should be developed, focusing not only on physical recovery but also integrating psychological support tailored to individual experiences and needs, assisting patients in gradually rebuilding a normal life (124). These programs are recommended to be designed by multidisciplinary teams, including psychiatrists, psychologists, and psychiatric nurses (125). Ongoing research is needed to evaluate the effectiveness of digital solutions and tailor them to the unique needs of long COVID patients (126). A combined approach of pharmacotherapy and psychological intervention is generally advised to address the multifaceted nature of LC. One study showed that combining cognitive behavioral therapy with pharmacotherapy (including non-benzodiazepine sedatives) significantly improved patients’ psychological health outcomes (8). The combination of NIBS and aerobic exercise therapy has also been used to improve the quality of life and reduce fatigue in LC patients (127).

With the continuous mutation of the novel coronavirus and the rising number of COVID-19 cases worldwide, many individuals have suffered from persistent long-term symptoms (128, 129). Notably, psychological symptoms often last longer than physical symptoms (130, 131). Research on psychological symptoms related to LC is gradually deepening, but many challenges remain, such as inconsistent diagnostic criteria, lack of long-term follow-up studies, and insufficient treatment strategies (43, 132). Given the prolonged duration and high prevalence of LC-related psychological symptoms, integrating psychological health care into comprehensive LC treatment plans is essential (133). Existing evidence suggests that psychotherapy, pharmacological interventions, and social support can effectively improve patients’ psychological health (134).

The psychological impact of LC is multidimensional and complex, involving a broad spectrum of symptoms, underlying pathophysiological mechanisms, and unique epidemiological characteristics. This review systematically summarized LC-associated psychological symptoms, including social stigma and shame, anxiety and depression, PTSD, fatigue, sleep disturbances, and cognitive dysfunction, all of which significantly reduce patients’ quality of life and social functioning. The underlying mechanisms may involve the interplay of direct viral invasion of the central nervous system, immune dysregulation and neuroinflammation, vascular dysfunction, HPA axis dysregulation, and psychosocial stress.

Current integrated interventions for these psychological symptoms include psychotherapy (e.g., cognitive behavioral therapy), pharmacotherapy (e.g., antidepressants), neuromodulation techniques (e.g., transcranial magnetic stimulation), hyperbaric oxygen therapy, lifestyle modifications (e.g., exercise and sleep management), and social support and rehabilitation programs. However, the long-term efficacy of existing treatments requires further validation, and the development of individualized intervention strategies is particularly important. Future research should further explore the deeper connections among these mechanisms to develop more personalized and refined intervention approaches, ultimately helping patients achieve better recovery.
